# Our City Physicians

**Published:** 1887-01-01

**Authors:** 


### OUR CITY PHYSICIANS.

The City Physicians of the city of Atlanta are, perhaps, the poorest paid of any city on the Continent. The services they render are, of course, among the lowest class of society, and are often most trying and laborious. Few things are more trying and more demoralizing than unremunerative labor. The reports which have been presented to the Council by the several Ward Physicians for the past year would, perhaps, if published, astonish the profession and place the city of Atlanta in no very enviable attitude as to the appreciation shown to their medical servants. We make brief allusion to one of these reports, kindly shown us by Dr. E. van Goidtsnoven, of the Second Ward. From this, it appears that the Doctor has treated, in his ward, during the year, whites, 304 ; colored, 166—making a total of 470. The number of visits made to the whites was 1,563, and of visits to the colored, 398—total, 1,961! Of these, there occurred one death among the whites and four among the colored. In the work here performed by the Doctor he was required to furnish the drugs used, which aggregated $70.50; and the compensation received amounted to only *twenty-two cents* a visit!

We note, with some surprise, that the diseases most prevalent in the city are the following, and in the order mentioned:

1. Nervous diseases.
2. Miasmatic diseases.
3. Digestive diseases.
4. Diathetic diseases.
5. Developmental diseases.

It is proper to state that, according to the Nosological table used, there were included under the Miasmatic head, croup, diarrhœa, dysentery, cholera infantum, measles, whooping-cough, mumps, diphtheria, etc.; the remittent, intermittent and typhoid fevers constituting only a share of those treated under this head. The small number of deaths which occurred speaks well for the skill and faithfulness of this poorly paid professional employee of a great city.

